# Investigation of Flow Boiling Heat Transfer Performance of Grooved Metal Foam (Ni, Cu) Evaporators

**DOI:** 10.3390/mi17030286

**Published:** 2026-02-25

**Authors:** Junteng Cao, Huajie Li, Xianbo Nian, Chaoyi Zhang, Yuankun Zhang, Chunsheng Guo

**Affiliations:** Centre for Omniscale Thermal Management and Comprehensive Energy Utilisation (OTM-EU), School of Airspace Science and Engineering, Shandong University, Weihai 264209, Chinanianxianbo@sdu.edu.cn (X.N.);

**Keywords:** high heat flux electronics cooling, metal foam, pore density, channel aspect ratio, boiling heat transfer

## Abstract

To meet the miniaturized cooling demands of high-heat-flux electronic devices, metal foams—featuring high specific surface area and multiscale porous structures—are considered promising candidates for enhancing flow boiling evaporation. However, pore density (PPI) and grooved geometry (channel aspect ratio, AR) jointly regulate vapor–liquid distribution, rewetting, and flow resistance, thereby constraining overall performance. Here, flow boiling experiments were conducted on nickel and copper foams with pore densities of 100, 500, and 1000 PPI and AR values of 0.7, 1.0, and 1.3. Heat transfer coefficient (HTC), wall superheat (ΔT), and pressure drop (Δp) were systematically evaluated, complemented by transient two-phase simulations revealing vapor fraction, temperature, and pressure drop distributions. A pronounced non-monotonic pore-density dependence is observed: 500 PPI achieves an optimal balance between heat-transfer enhancement and flow resistance, whereas 100 PPI suffers from vapor accumulation and temperature non-uniformity, and 1000 PPI is penalized by excessive permeability resistance and pore-scale confinement. An optimal AR of 1.0 promotes efficient vapor venting and stable rewetting. Under the optimal configuration (500 PPI, AR =1.0), a limiting heat flux of 348.6 W/cm^2^, corresponding to the HTC of 55.4 kW/(m^2^ · K), and a limiting HTC of 130.3 kW/(m^2^ · K) are achieved, providing quantitative design guidelines for metal-foam two-phase evaporators.

## 1. Introduction

The continuous development of AI-driven computing paradigms, such as deep learning inference and real-time data analytics, has pushed the computational workload of edge data centers to unprecedented levels [1]. The resulting high board-level heat flux densities fundamentally challenge the effectiveness of existing cooling approaches in maintaining device reliability and operational stability [2]. Alami et al. [3] numerically demonstrated that the heat transfer efficiency of natural convection air cooling in horizontal channels is strongly correlated with heater spacing, revealing the dynamic balance characteristics of air cooling under low heat flux conditions (<$3\;\mathrm{kW}/m^{2}$). A survey by Zhang et al. [4] reported that conventional air cooling can typically handle up to $1.55\;\mathrm{kW}/m^{2}$ under natural convection, and only about $100\;W/{\mathrm{cm}}^{2}$ even under forced convection. In particular, due to the poor thermophysical properties of air together with undesirable noise and vibration, air cooling exhibits inherent limitations in high heat flux applications.

In addition, single-phase liquid cooling relies primarily on sensible heat transfer, and further performance enhancement usually requires increased coolant flow rates, reduced channel dimensions, or intensified flow disturbances. These measures inevitably lead to a rapid increase in pumping power and render system performance highly sensitive to flow distribution and operational control. As thermal loads continue to rise and system nonlinearity becomes more pronounced, single-phase thermal management systems operating near their performance limits exhibit an increasingly strong dependence on accurate sensing, system calibration, and model fidelity. Recent studies have shown that, under complex operating conditions, in situ sensor calibration methods based on virtual samples and autoencoder frameworks are required to effectively mitigate measurement uncertainty and maintain reliable system [5]. Furthermore, virtual sample diffusion methods guided by large language model-generated knowledge have been proposed to enhance information completeness and support system diagnosis and performance evaluation under data-scarce or previously unseen operating [6]. These studies collectively indicate that single-phase cooling systems inherently depend on modeling accuracy and control strategies when operating under high heat flux and complex conditions.

In contrast, two-phase cooling exploits the large latent heat associated with liquid–vapor phase transition, enabling efficient heat removal at an approximately constant saturation temperature. This allows temperature non-uniformity and hot-spot formation to be suppressed without relying on extreme flow rates or excessive pumping power, thereby offering superior scalability and energy efficiency. Indirect liquid cooling approaches (e.g., liquid-cooled cold plates with circulating coolant) and direct liquid cooling methods (e.g., immersion and spray cooling) have demonstrated excellent thermal management capability in commercial applications, such as Microsoft’s Project Natick underwater data center. With the development of fluorinated working fluids featuring precisely tunable boiling points and low viscosity, Fallahtafti et al. [7] combined geometric modification (e.g., cutting fins to form vertical microchannels) with neural network modeling and optimization, demonstrating that a commercial two-phase cold plate using HFE-7000 can achieve a heat flux as high as $73.5\;W/{\mathrm{cm}}^{2}$. Wang et al. [8] investigated immersion phase-change cooling using SF33 for 18,650 lithium-ion batteries and maintained the cell temperature below 34 °C (0.5 °C below the boiling point), with a temperature difference less than 5 °C under high-rate discharge and dynamic load conditions. Qin et al. [9] proposed a closed-loop integrated system based on $C_{6}F_{12}O$ spray cooling, in which spray cooling absorbs approximately 70% of the total heat dissipation and significantly suppresses thermal runaway. Nevertheless, compared with optimized two-phase cooling systems, these approaches still face challenges such as high maintenance costs and substantial pumping power consumption.

In recent years, passive two-phase cooling technologies, including micro loop heat pipes [10,11] and vapor chambers [12,13], have continued to evolve for on-chip cooling applications. Although effective under steady-state conditions, these systems exhibit significant limitations in mitigating transient thermal shocks and sustaining ultra-high heat flux densities (>$300\;W/{\mathrm{cm}}^{2}$), primarily due to insufficient capillary driving force and limited responsiveness to abrupt heat load variations. In this context, mechanically pumped two-phase loop (MPTL) systems have attracted increasing attention because they can leverage phase-change heat transfer to improve cooling efficiency under high heat flux conditions. Erp et al. [14] fabricated a microfluidic manifold microchannel cooling structure integrated with electronic devices, achieving a cooling performance of up to $1700\;W/{\mathrm{cm}}^{2}$. These results highlight the great potential of combining micro/nanostructures with mechanically pumped two-phase systems for high-heat-flux electronics thermal management. However, manufacturing complexity and high cost remain major barriers to large-scale industrial implementation.

Metal foams, characterized by high porosity, controllable geometry, large specific surface area, and strong thermal conductivity, have received considerable attention in two-phase enhanced heat transfer applications [15]. When coupled with capillary effects, metal foams facilitate rapid bubble departure and enhanced liquid replenishment, thereby improving boiling flow stability [16]. In addition, their lightweight nature makes them particularly attractive for weight-sensitive applications [17]. Regarding evaporative heat dissipation using metal foams, Manetti et al. [18] investigated pool-boiling heat transfer of HFE-7100 on copper and nickel foams and analyzed bubble dynamics using high-speed imaging. Their results indicated that when the heat flux exceeds $120\;\mathrm{kW}/m^{2}$, the large surface area of metal foams restricts bubble escape pathways, leading to local vapor accumulation and increased resistance to bubble departure. Topin et al. [19] measured pressure distributions and conducted Forchheimer-model analyses on copper foams with pore densities ranging from 10 to 100 PPI, concluding that low-PPI foams reduce pressure drop but provide limited enhancement in flow boiling heat transfer. Kim et al. [20] investigated flow boiling heat transfer of R245fa in a rectangular channel filled with 18 PPI open-cell copper foam, reporting that the foam significantly increases bubble nucleation and departure rates at low vapor quality and further enhances heat transfer at high vapor quality by strengthening liquid–vapor interactions. Moreover, Mancin et al. [21] systematically examined the effects of porosity, pore density, and foam thickness on heat transfer performance and found that copper foam outperforms aluminum foam in heat dissipation. Hu et al. [22] studied the effects of pore size, heat flux, and capillary force on bubble escape in porous evaporators, showing that smaller pore sizes (50μm) lead to higher bubble departure frequency and the formation of continuous vapor-venting pathways, thereby improving heat transfer efficiency. Hsu et al. further investigated the coupled effects of channel geometric parameters on two-phase flow boiling heat transfer performance and pressure drop, emphasizing the critical role of geometric confinement on flow stability under high heat flux [23]. At the pore scale level, previous studies have analyzed vapor–liquid phase distribution and bubble dynamics in porous or metal foam-based evaporators, elucidating the role of local phase change processes in heat transfer [24]. Other studies have systematically discussed the influence of geometric confinement on flow boiling heat transfer and pressure drop [25]. In addition, experimental and numerical investigations have revealed the mechanisms of vapor retention and two-phase flow resistance in porous media, explaining heat transfer degradation under high vapor quality [26].

Although the aforementioned studies have deepened the understanding of flow boiling behavior in metal foam-filled microchannels from different perspectives, most of them focus on individual structural factors or localized heat transfer mechanisms. Moreover, existing research has primarily concentrated on low PPI metal foams, while flow boiling characteristics in high PPI (≥100) foams remain relatively underexplored. Systematic investigations at the engineering evaporator scale that simultaneously consider high porosity metal foams and channel geometric parameters are still lacking, which motivates the present study. High PPI grooved metal foams establish an effective vapor–liquid separation mechanism that preserves excellent heat transfer performance while mitigating excessive flow resistance. In particular, the channel aspect ratio (AR) directly affects geometric confinement in bubble growth and migration, interfacial shear, and rewetting processes in confined spaces, thereby determining heat transfer coefficient enhancement, pressure drop growth rate, and boiling stability [27,28]. Therefore, building upon prior studies and targeting high-heat-flux electronics cooling applications, the present work conducts systematic experimental investigations of grooved metal foam evaporators. By employing nickel and copper foams with different pore densities and constructing internal transport channels with varying AR, the heat transfer coefficient (HTC), wall superheat (ΔT), and performance evaluation coefficient (PEC) under flow boiling conditions are systematically evaluated, revealing the dominant roles of pore density and channel geometry in comprehensive heat dissipation performance and providing design guidance for evaporator structures with both high heat transfer capability and low flow resistance.

## 2. Experimental Apparatus

### 2.1. Experimental Setup

**Figure 1:**
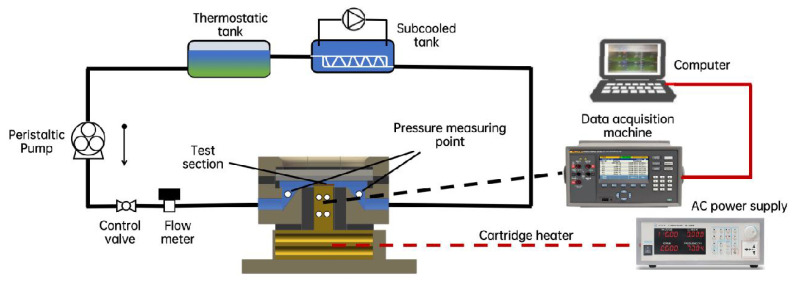
Flow boiling experimental system.

A flow boiling experimental system was constructed (Figure 1), consisting of an evaporation platform, thermostatic tank, subcooled tank, peristaltic pump (KPP-DA-B08W, Kamoer, Shanghai, China), cartridge heater, flow meter, pressure sensors, power supply (APS400B, IVYTECH, Indianapolis, IN, USA), and data acquisition system (2638A/20, FLUKE, Everett, WA, USA). The evaporator heating component is an oxygen-free copper heating block equipped with eight A-grade cartridge heaters (diameter 6 mm and length 40 mm), with a total power of 960 W. Accordingly, a PS-6101 power supply (Bosch Rexroth, Lohr am Main, Germany) was used; with doubled voltage enabled, it can provide a maximum power of 1000 W, which meets the experimental requirement, with power fluctuation below 1 W during testing. Six PT100 (WIKA, Klingenberg, Germany) (accuracy ±0.1 °C) were arranged in layers to monitor temperature distribution in real time.

A MIK-P300G diffused-silicon high-temperature pressure transducer (Hangzhou Meacon, Hangzhou, China) was powered by 24 V DC using a Delixi 220 V–24 V switching power supply (Delixi, Shanghai, China). Data were recorded using a paperless recorder (MIK-R6000C, Hangzhou, China). The pressure sensor range is 0–100 kPa, suitable for pressure measurements during experiments. Metal foam samples were grooved by wire electrical discharge machining (WEDM) and reliably soldered to the heating block surface using high-temperature lead-free solder (Sn99Ag0.3Cu0.7). The detailed parameters and process flow for wire cutting of foam metal using electric discharge machining are provided in Supplementary Note S1. The detailed welding process and description of thermal resistance at the welding point are provided in Supplementary Note S2. The working fluid was deionized water at 60 °C, operated at atmospheric pressure and an ambient temperature of 27 ± 1 °C. Deionized water was selected in this study for two main reasons. First, to meet the junction-temperature control requirements of SiC power devices (about 150 °C), water can still provide robust thermal management in an atmospheric-pressure loop (with a saturation temperature of about 100 °C): under high heat flux conditions, its high latent heat of vaporization enables stronger phase change heat absorption and more stable heat dissipation performance, thereby supporting the thermal control objectives for high temperature electronics. Second, because the experiments were conducted in an atmospheric-pressure recirculating loop, deionized water serves as a representative baseline fluid that maintains strong repeatability while reducing uncertainties associated with coupling between fluid properties and system pressure, allowing the structural effects of pore density (PPI) and groove aspect ratio (AR) on HTC, CHF, and pressure drop to be more clearly isolated and compared. All thermocouple signals were automatically recorded at 0.5 s intervals, and the steady state was identified when the temperature variation within 1 min did not exceed 0.5 °C. When there is a sudden increase in temperature, and it enters an irreversible upward trend, a drying instability is identified, and CHF is considered exceeded if the temperature at any measurement point rises by more than 20 °C within 10 s and continues to rise or fails to return to the original quasi-steady fluctuation band within the subsequent 30 s.

### 2.2. Evaporator Structure and Sample Design

**Figure 2:**
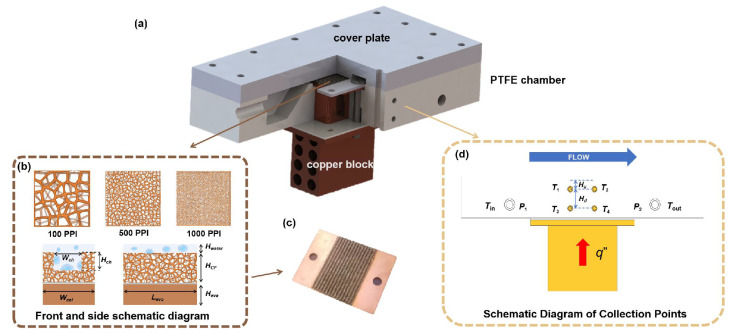
Schematics of evaporator and samples with varying PPIs: (**a**) An integrated evaporator structure. (**b**) The front and side schematic diagrams of the metal foam structures with different pore densities (100, 500, and 1000 PPI). (**c**) A representative grooved copper base with embedded metal foam. (**d**) The data acquisition and processing scheme.

As shown in Figure 2a, an integrated evaporator structure was adopted. The metal foam sample was embedded in a grooved cavity of a copper base and sealed by an upper cover plate and PTFE chamber to form a closed flow channel. The copper block beneath the base serves as the heating interface. The working fluid enters the evaporation region along the flow direction (FLOW), where two-phase flow and phase-change heat transfer occur. Figure 2b presents the front and side schematic diagrams of the metal foam structures with different pore densities (100, 500, and 1000 PPI), highlighting the geometric parameters of the channel, including the channel width ($W_{\mathrm{ch}}$), channel height ($H_{\mathrm{ch}}$), and foam thickness. These parameters define the controllable vapor-venting and liquid-replenishment pathways within the grooved cavity. Figure 2c shows a photograph of a representative grooved copper base with embedded metal foam, illustrating the actual fabricated structure used in the experiments. The data acquisition and processing scheme is illustrated in Figure 2d, where the effective heat flux $q^{\prime \prime }$ is applied at the bottom of the copper base, and temperature and pressure measurement points are arranged along the flow direction. The inlet and outlet temperatures ($T_{\mathrm{in}}$ and $T_{\mathrm{out}}$) and pressure drop (Δp) are measured to calculate the heat transfer coefficient (HTC) and further evaluate the performance evaluation coefficient (PEC).

The copper base provides both heat conduction and structural support: external heating is applied at the bottom of the base, and heat is conducted through the copper block into the metal foam, then removed by the working fluid, forming a coupled heat transfer pathway of solid conduction, convection, and phase-change evaporation. The sample design focuses on two groups of variables: pore density (PPI), representing differences in foam ligament scale and connectivity, and channel aspect ratio (defined by $W_{\mathrm{ch}}$ and $H_{\mathrm{ch}}$). Thus, the cooling performance under different structural parameters can be compared within a unified quantitative framework. The various structural parameters are summarized in Table 1.

## 3. Data Detection and Uncertainty Analysis

### 3.1. Data Reduction

The effective heat flux, $q^{\prime \prime }$, was calculated according to the deformation of Fourier’s law, which can be expressed as follows:

(1) $$q^{\prime \prime }=\frac{k_{\mathrm{OTC}}\left(T_{3}+T_{4}-T_{1}-T_{2}\right)}{2L_{2}}$$

where $k_{\mathrm{OTC}}$ represents the thermal conductivity of the OFC block. The value of $k_{\mathrm{OTC}}$ is set to 400W/(m·K). $L_{2}$ (m) is the distance between the upper and lower temperature measurement points of the heating block, taken as 10 mm. In this experiment, if the system fails to stabilize, the heat dissipation of the evaporator is considered to have reached its limit, and the corresponding heat flux density is defined as the critical heat flux (CHF).

The boiling convective heat transfer coefficient (HTC) can be calculated as follows:

(2) $$h=\frac{q^{\prime \prime }}{T_{w}-T_{\mathrm{sat}}}$$

where $T_{w}$ (K) represents the temperature at the bottom of the CFs, while $T_{\mathrm{sat}}$ (K) denotes the saturated temperature of the deionized water. According to Fourier’s law, the temperature difference between the upper and lower thermocouples was proportionally extrapolated to estimate the bottom surface temperature of NFs/CFs. Given that $T_{w}$ may be lower than the saturation temperature during the subcooling or initial boiling stage with low vapor quality, a segmented function was adopted for determining $T_{\mathrm{sat}}$, which can be expressed as follows:

(3) $$T_{w}=\frac{T_{1}+T_{2}}{2}-\frac{L_{1}}{L_{2}}\left[\frac{T_{3}+T_{4}}{2}-\frac{T_{1}+T_{2}}{2}\right]$$

(4) $$T_{\mathrm{sat}}=\left\{\begin{array}{cc} T_{\mathrm{out}}, & T_{w}\leq 373.15\;K\\ 373.15\;K, & T_{w}>373.15\;K \end{array}\right.$$

where $L_{1}$ (m) represents the distance between the upper measurement points and the bottom of the NF/CF samples, taken as 10 mm. Additionally, the heat dissipation capability of the sample was evaluated by the superheat ΔT, which can be expressed as follows:

(5) $$\Delta T=T_{w}-T_{\mathrm{sat}}$$

The positions of $T_{\mathrm{in}}$ and $T_{\mathrm{out}}$ are shown in Figure 2d.

The pressure drop is defined as the static pressure difference between the inlet and outlet pressure taps across the following test section:

(6) $$\Delta p=P_{2}-P_{1}$$

The positions of $P_{1}$ and $P_{2}$ are shown in Figure 2d, where $P_{1}$ and $P_{2}$ are synchronously recorded by two pressure transducers. For each heat flux step, the pressure drop signal is processed using the same quasi-steady criterion and time-averaging procedure as the temperature data; specifically, once quasi-steady conditions are reached, Δp is obtained by averaging over a continuous 1 min window. The pressure drop used in the PEC evaluation is exactly the Δp defined and processed above, ensuring traceability and consistency of the thermo-hydraulic assessment.

### 3.2. Uncertainty Analysis

(1) Uncertainty in Temperature Measurement. The thermocouples used in this experiment have a measurement range of 0–800 °C, with an individual measurement error of ± 0.5 °C. The lowest temperature recorded in the experiment is 75 °C, and the uncertainty in temperature measurement is calculated as

(7) $$U\left(T\right)=\frac{0.5}{75}\times 100\%=0.67\%$$

(2) Uncertainty impact of $T_{w}$ on HTC. Because the wall temperature $T_{w}$ is obtained by extrapolating from temperature sensors embedded inside the heating block, its sensitivity to the sensor location uncertainty Δz can be estimated using a one-dimensional conduction approximation:

(8) $$\Delta T_{w}\approx \frac{q^{\prime \prime }\;\Delta z}{k}$$

In this work, the heating block is oxygen-free copper (OFC) with k=400W/(m·K). Considering machining and installation tolerances for the embedded sensors (PT100), a conservative location uncertainty of Δz=0.1–0.2mm is assumed. For a representative high heat flux condition in this study, $q^{\prime \prime }=350\;W/{\mathrm{cm}}^{2}$, the resulting extrapolation uncertainty is

(9) $$\Delta T_{w}\left(\Delta z=0.1\;\mathrm{mm}\right)\approx 0.87\;K$$

(10) $$\Delta T_{w}\left(\Delta z=0.2\;\mathrm{mm}\right)\approx 1.74\;K$$

Therefore, at the highest heat flux level of this study, the extrapolation uncertainty introduced solely by sensor location is typically within about 0.9–1.7 K. When combined with the temperature measurement accuracy (±0.1 °C) and the uncertainty associated with gradient fitting, the absolute uncertainty of $T_{w}$ can reasonably be considered to be on the order of about 1–2 K. This uncertainty propagates into the HTC through Equation (2). The relative uncertainty of HTC can be approximated by

(11) $${\left(\frac{u(h)}{h}\right)}^{2}\approx {\left(\frac{u(q^{\prime \prime })}{q^{\prime \prime }}\right)}^{2}+{\left(\frac{u(\Delta T)}{\Delta T}\right)}^{2}$$

For the typical superheat range in this work, ΔT≈20–60K, if $u(T_{w})\approx 1$–2K, the contribution from the temperature difference term is approximately 1.7–10%. Accordingly, the absolute uncertainty in HTC associated with the temperature difference term is on the order of several percent up to about 10%.

(3) Uncertainty in Pressure Measurement. The uncertainty of Δp is estimated based on the pressure transducer accuracy. The transducers have a full-scale range of 0–50 kPa with an accuracy class of 0.075, corresponding to ±0.075% of full-scale. Thus, the maximum indicated error for a single transducer is

(12) δp=0.075%×50kPa=0.0375kPa

Assuming a rectangular distribution, the Type-B standard uncertainty of a single pressure reading is

(13) $$u\left(p\right)=\frac{\delta p}{\sqrt{3}}=0.0217\;\mathrm{kPa}$$

Assuming the two transducers have identical accuracy and are statistically independent, the combined standard uncertainty of the pressure drop is

(14) $$u\left(\Delta p\right)=\sqrt{u{\left(p_{1}\right)}^{2}+u{\left(p_{2}\right)}^{2}}=0.0306\;\mathrm{kPa}$$

(4) Uncertainty in Power Supply Measurement. The uncertainty associated with the DC power supply and related components is determined using the Type-B uncertainty formula, expressed as

(15) $$U\left(E\right)=\frac{O}{K}$$

where *O* represents the nominal error specified in the instrument manual. The nominal error is given as ±1%, resulting in a power supply uncertainty of 0.58%. The uncertainty in the input heat load is calculated using

(16) $$U\left(Q_{\mathrm{in}}\right)=\frac{\sqrt{{\left(I\cdot u(V)\right)}^{2}+{\left(V\cdot u(I)\right)}^{2}}}{V\cdot I}$$

The calculation results indicate that the error remains within an acceptable range.

## 4. Results and Discussion

### 4.1. Pore Density

**Figure 3:**
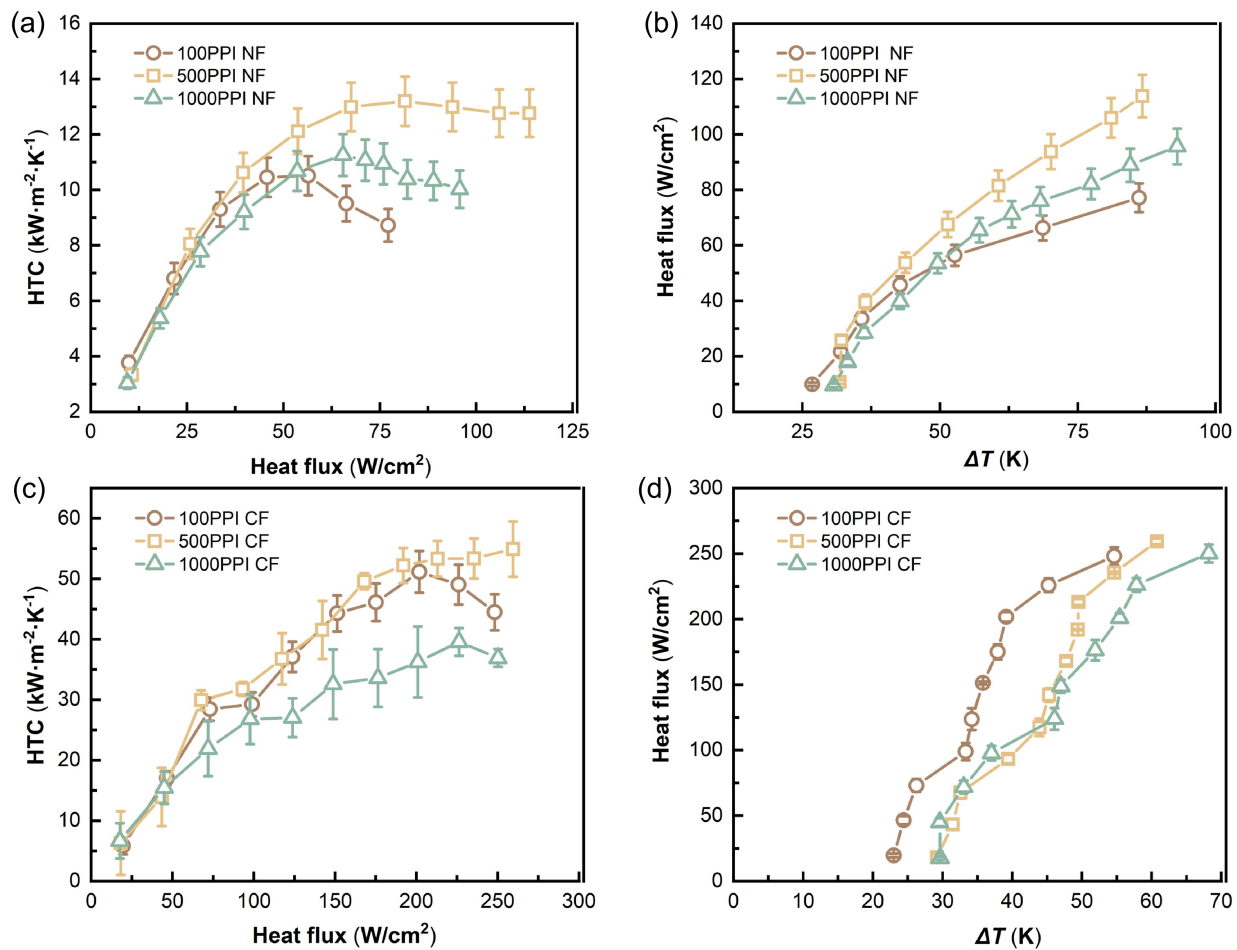
HTC–$q^{\prime \prime }$ and $q^{\prime \prime }$–ΔT curves for ungrooved metal foams: (**a**) HTC–$q^{\prime \prime }$ for NFs with different PPI. (**b**) $q^{\prime \prime }$–ΔT for NFs with different PPI. (**c**) HTC–$q^{\prime \prime }$ for CFs with different PPI. (**d**) $q^{\prime \prime }$–ΔT for CFs with different PPI.

As shown in Figure 3a,b, ungrooved NFs exhibit pronounced differences in flow boiling heat transfer under different pore densities. With increasing heat flux $q^{\prime \prime }$, the HTCs of all three samples rise rapidly to a peak. However, the 500 PPI curve maintains a relatively stable high HTC after reaching its peak (about 12–13 kW/(m^2^ · K)), indicating strong sustained heat transfer capability. The 100 PPI sample increases rapidly at low heat flux but shows an earlier decline at higher $q^{\prime \prime }$, because the larger pore structure is more prone to adverse effects under intense vaporization, such as local vapor accumulation and limited liquid renewal [29]. The 1000 PPI sample is overall lower than the 500 PPI case, indicating that excessive pore density, associated with higher flow resistance and pore-scale confinement, can weaken effective liquid replenishment and vapor removal [30,31]. Consistent with the HTC–$q^{\prime \prime }$ trend, the $q^{\prime \prime }$–ΔT relationship (Figure 3b) shows that, at the same wall superheat, 500 PPI achieves a higher heat flux output, further demonstrating better cooling performance.

As shown in Figure 3c,d, ungrooved CFs, due to higher thermal conductivity and stronger heat spreading capability, exhibit substantially higher HTC levels than NFs and can sustain enhanced boiling over a higher heat flux range. The three curves also show a rapid rise followed by a plateau or decay. Among them, 500 PPI achieves the highest HTC (about 50–55 kW/(m^2^ · K)) in the high heat flux regime and maintains higher heat flux output at larger superheat (>55 K). The 100 PPI sample can approach the 500 PPI case at moderate heat flux but shows earlier decay at higher $q^{\prime \prime }$. The 1000 PPI sample remains lower overall, reflecting that at high vaporization intensity, higher pore density tends to increase flow resistance and intensify local two-phase retention, thereby limiting effective heat transfer. Figure 3d shows that at the same ΔT in the low-to-moderate heat flux range, the 100 PPI curve generally corresponds to higher $q^{\prime \prime }$, but it cannot reach very high heat flux.

To comprehensively evaluate the effect of PPI on flow boiling heat dissipation enhancement, the performance evaluation coefficient (PEC) is introduced. PEC integrates heat transfer and pressure drop to assess the overall performance of single- or two-phase evaporators:

(17) $$\mathrm{PEC}=\frac{h/h_{\mathrm{ref}}}{{\left(\Delta p/\Delta p_{\mathrm{ref}}\right)}^{1/3}}$$

where *h* is the heat transfer coefficient and Δp is the pressure drop in the test section. The subscript “ref” denotes the reference baseline condition; for pore density (PPI), the reference condition is 100 PPI. When PEC>1, the overall thermo-hydraulic performance is improved relative to the reference.

**Figure 4:**
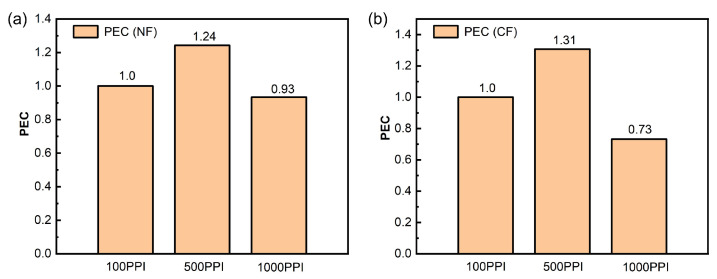
(**a**) PEC of NFs with different PPI (100 PPI represents the standard condition). (**b**) PEC of CFs with different PPI (100 PPI represents the standard condition).

As shown in Figure 4, the PEC of 500 PPI NF increases to 1.24, significantly higher than that of 100 PPI, whereas the PEC of 1000 PPI decreases to 0.93. For CFs, the PEC of 500 PPI increases to 1.31, significantly higher than that of 100 PPI, while the PEC of 1000 PPI decreases to 0.73. These results indicate that although higher pore density may provide more nucleation-related structures, the smaller pore scale and stronger tendency toward two-phase blockage can markedly increase flow resistance, thereby offsetting or even reversing heat transfer gains. In other words, the reason 500 PPI becomes optimal is that it achieves a more reasonable balance between enhanced nucleation and acceptable flow resistance. Discussion on Δp is provided in Supplementary Note S3. It should be emphasized that the superiority of 500 PPI reported here is identified within the tested parameter space and operating conditions of this study (deionized water, atmospheric-pressure loop, and the investigated mass flux and heat flux ranges). The optimal PPI may shift when the working fluid, flow rate, inlet subcooling, system pressure, or heating boundary condition changes; therefore, 500 PPI should be regarded as a case-specific design reference rather than a universal optimum.

**Figure 5:**
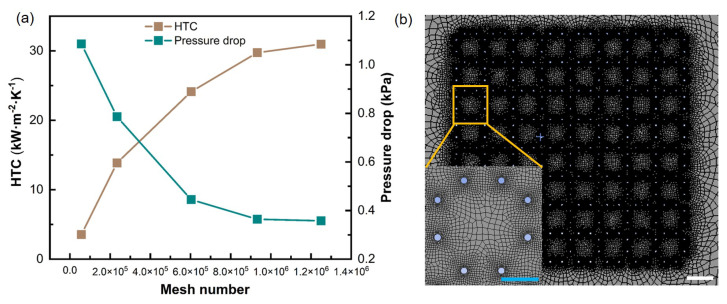
(**a**) Grid-independence results for the 500 PPI case at $q^{\prime \prime }=80\;W/{\mathrm{cm}}^{2}$. (**b**) Schematic diagram of the computational-domain grid and local refinement details. White scale bar: 0.16 mm; blue scale bar: 0.06 mm.

To ensure reliable transient two-phase VOF simulations in resolving interfacial evolution and predicting HTC and pressure drop, a geometry-feature-based non-uniform meshing strategy was employed, and the final mesh density was determined via a grid-independence study. Grid-independence was evaluated for a representative case (500 PPI, $q^{\prime \prime }=80\;W/{\mathrm{cm}}^{2}$), as shown in Figure 5a. With increasing mesh number, both HTC and Δp converge; when the total cell number reaches $9.32\times 10^{5}$, the simulated HTC converges to 29.74, and Δp converges to 0.36 kPa, and further refinement produces only marginal changes. Therefore, a mesh of $9.32\times 10^{5}$ cells was adopted for all subsequent simulations as a compromise between numerical accuracy and computational cost.

Figure 5b shows the mesh schematic: the computational domain includes the equivalent CF region and the surrounding flow channel, and an unstructured quad-dominant mesh was generated with smooth size transition. Local refinement was applied around the equivalent foam features and near the grooved channel walls, where strong phase-interface variation and steep thermal or hydrodynamic gradients are expected, while relatively coarser cells were used in regions away from these features to reduce computational cost.

**Figure 6:**
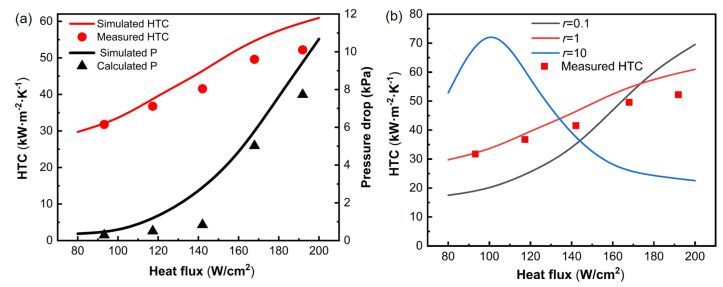
(**a**) Comparison between simulations and experiments for the HTC and Δp of the 500 PPI copper foam under various heat fluxes. (**b**) Sensitivity analysis of the Lee phase-transition model parameter *r*.

To validate the credibility of the transient two-phase numerical simulations, the model was benchmarked against experimental measurements and a sensitivity analysis of the Lee phase transition model parameter *r*. Figure 6a compares the experimentally measured and numerically predicted heat transfer coefficient (HTC) and pressure drop (Δp) over a range of heat fluxes. The simulated HTC increases monotonically with increasing heat flux, exhibiting the same trend and comparable magnitude to the experimental data, indicating that the proposed two-phase heat transfer model can reproduce the primary experimental behavior. In addition, a representative operating condition was selected for quantitative validation: a 500 PPI copper foam at a heat flux of $q^{\prime \prime }=160\;W/{\mathrm{cm}}^{2}$. Under this condition, the simulation predicts $HTC_{\mathrm{sim}}=52.78\;\mathrm{kW}/\left(m^{2}\cdot K\right)$, while the experimentally measured value $HTC_{\exp}$ is about $49.59\;\mathrm{kW}/(m^{2}\cdot K)$, corresponding to a relative deviation of approximately 6.43%, demonstrating strong quantitative agreement in heat transfer intensity. For the same condition, the simulated pressure drop is $\Delta p_{\mathrm{sim}}=4.52\;\mathrm{kPa}$ compared to the experimental value $\Delta p_{\exp}=5.02\;\mathrm{kPa}$, resulting in an absolute difference of 0.50 kPa; the magnitude is comparable, and the model captures the pressure drop level. It should be noted that Δp is more sensitive to inlet and outlet local losses, the absence of inherently three-dimensional disturbance or secondary flows in a two-dimensional approximation, and the tortuosity and connectivity of the foam structure; therefore, the discrepancy in Δp is generally larger than that in HTC. Nevertheless, since the simulations are primarily used to ensure trend consistency and to support mechanistic interpretation, this deviation does not affect the conclusions regarding vapor–liquid distribution and the heat transfer mechanisms.

Figure 6b evaluates the sensitivity of the predicted HTC to the mass transfer coefficient *r* in the Lee phase-change model. Three representative values (r=0.1, 1, and 10) were tested while keeping all other physical models and boundary conditions unchanged. The results show that *r* exerts a direct control on the interfacial phase-change rate and therefore on the vapor generation intensity. When a small coefficient (r=0.1) is used, the phase-change source term becomes weak, leading to delayed vapor formation and an underestimation of the boiling enhancement, especially at moderate-to-high heat fluxes. In contrast, an excessively large coefficient (r=10) accelerates phase change and promotes premature vapor generation; this produces an unrealistically high HTC at low-to-moderate heat fluxes and subsequently an earlier vapor-coverage tendency, which suppresses the HTC at higher heat fluxes and yields a non-monotonic response that deviates from the experimental trend. Among the tested values, r=1 provides the best agreement with the experimental HTC over the entire heat flux range, reproducing both the monotonic increase and the correct magnitude. Therefore, r=1 was selected as the baseline value for the subsequent simulations, as it represents a balanced vapor-generation rate and avoids over- or under-prediction associated with extreme *r* values. A more detailed *r* assessment will be conducted in subsequent studies. This sensitivity study indicates that, although the Lee model contains an empirical constant, the main conclusions drawn in this work are supported by a parameter choice that is explicitly calibrated against experimental measurements and verified to yield physically consistent trends.

Overall, the numerical framework was validated from the following two complementary aspects:

(1) Direct benchmarking against the experimental data shows that the model captures both the trend and magnitude of the HTC and predicts the pressure drop at a comparable order of magnitude, demonstrating a reasonable thermo-hydraulic predictive capability (Figure 6a).

(2) The grid-independence study confirms numerical convergence, while the sensitivity analysis of the Lee phase-change mass transfer coefficient *r* further demonstrates that the selected parameter provides the best agreement with the experiments and avoids unphysical vapor-generation behavior (Figure 6b). Collectively, these validations support the credibility of the transient two-phase numerical simulations.

Finally, because metal foams inherently possess three-dimensional connectivity and multiscale tortuous pores, a two-dimensional VOF framework cannot fully represent the true three-dimensional bypass pathways, local phase trapping, and the influence of tortuosity on pressure drop and phase distribution. Therefore, the present two-dimensional simulations are used primarily to capture the qualitative evolution of the vapor–liquid interface at the channel scale, the relative distributions of temperature and pressure fields, and the experimentally consistent trends, rather than serving as a strict quantitative surrogate for the three-dimensional foam microstructure, thereby avoiding over-interpretation of the numerical results.

**Figure 7:**
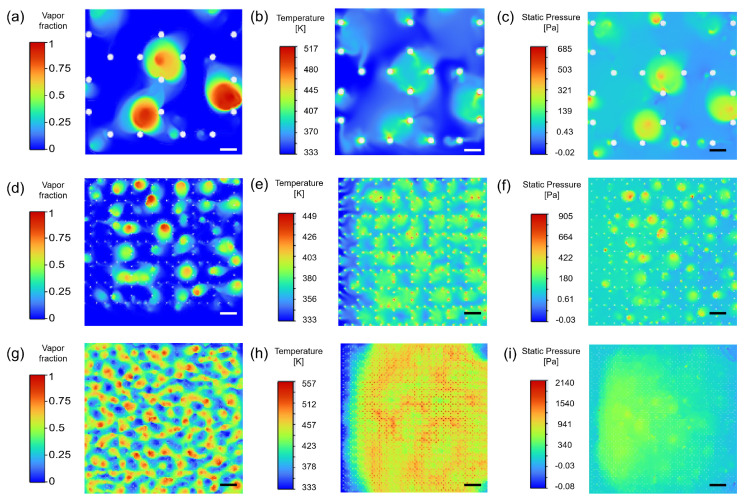
Vapor volume fraction, temperature, and pressure drop contours under different PPI: (**a**) vapor volume fraction at 100 PPI; (**b**) temperature at 100 PPI; (**c**) pressure drop at 100 PPI; (**d**) vapor volume fraction at 500 PPI; (**e**) temperature at 500 PPI; (**f**) pressure drop at 500 PPI; (**g**) vapor volume fraction at 1000 PPI; (**h**) temperature at 1000 PPI; (**i**) pressure drop at 1000 PPI. Scale bar: 0.15 mm.

To further support the heat dissipation advantage of 500 PPI and summarize the gas-phase distribution characteristics under different pore densities, a transient two-phase flow boiling model in ANSYS Fluent 2022 R1 was used to compare pore density cases (100, 500, and 1000 PPI). The detailed simulation settings are provided in Supplementary Note S4. As shown in Figure 7a–i (a–c: 100 PPI; d–f: 500 PPI; g–i: 1000 PPI), pore density alters pore scale and ligament connectivity, directly reshaping the vapor volume fraction distribution (phase field), temperature field, and pressure drop characteristics within the foam, thereby yielding different balances between heat transfer enhancement and flow resistance.

For 100 PPI (Figure 7a–c), the vapor phase mainly appears as a few large-scale vapor agglomerates (distinct red high-volume-fraction regions with small counts), indicating that bubbles in larger pores are more likely to coalesce and grow into locally covering vapor clusters. Correspondingly, the temperature contour exhibits stronger local non-uniformity: high-temperature regions often coincide with areas adjacent to vapor clusters, suggesting restricted rewetting and a higher tendency for localized heat accumulation. The pressure contour shows a relatively low overall magnitude (colorbar upper limit of about 685 Pa), but local pressure disturbances aligned with vapor-cluster distribution are observable, reflecting intermittent disturbances induced by vapor accumulation.

By contrast, 500 PPI (Figure 7d–f) exhibits field distributions more consistent with efficient heat dissipation. The vapor field shows a larger number of smaller vapor structures with a more uniform spatial distribution, enabling continuous phase-change activity while avoiding the large-cluster-induced local coverage and rewetting degradation observed at 100 PPI. The temperature field is more uniform with a lower peak (colorbar upper limit of about 449 K), and hotspots are clearly suppressed, indicating more stable liquid renewal and two-phase heat transfer. The pressure level remains controllable (colorbar upper limit of about 906 Pa), and the pressure gradient is smoother, suggesting that effective vapor venting is achieved without excessive flow resistance penalties.

For 1000 PPI (Figure 7g–i), the vapor field becomes denser, with high vapor volume fraction structures widely distributed across the domain; under smaller pore-scale confinement, vapor tends to be retained. The temperature contour shows a larger fraction of high-temperature area and the highest peak (colorbar upper limit of about 557 K), indicating difficulty in maintaining uniform cooling when liquid replenishment is constrained. Meanwhile, the pressure magnitude increases markedly (colorbar upper limit of about 2140 Pa), reflecting higher intrinsic flow resistance associated with high pore density. Overall, 500 PPI forms a synergistic advantage in terms of controllable vapor distribution, more uniform and lower peak temperature, and moderate pressure drop, thereby providing a more direct explanation for its superior experimental performance. Based on this conclusion, it can be further inferred that if flow channels can be structurally optimized (e.g., by comparing different channel aspect ratios) while preserving the advantages in vapor venting and rewetting, the 500 PPI sample still has potential to further enhance stability and overall heat dissipation capability under high heat flux.

### 4.2. Channel Aspect Ratio

**Figure 8:**
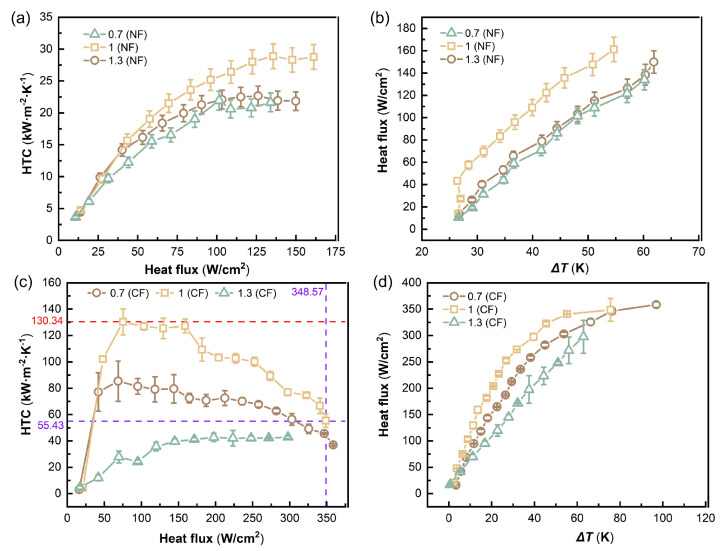
(**a**) HTC–$q^{\prime \prime }$ for NF at different AR. (**b**) $q^{\prime \prime }$–ΔT for NF at different AR. (**c**) HTC–$q^{\prime \prime }$ for CF at different AR. (**d**) $q^{\prime \prime }$–ΔT for CF at different AR.

As shown in Figure 8a,b, for NF at the same pore density (500 PPI), the channel aspect ratio (AR = 0.7, 1.0, and 1.3) significantly changes the convective heat transfer level and its evolution with increasing heat flux. Overall, HTC increases rapidly with $q^{\prime \prime }$ and then enters a relatively steady or slowly increasing regime at moderate-to-high heat flux. However, the plateau level and stability vary markedly with AR. AR = 1.0 remains at a higher level in the mid-to-high heat flux region and shows stronger stability, while AR = 0.7 and AR = 1.3 exhibit lower HTC values and poorer stability than AR = 1.0. This indicates that when AR is close to unity, the channel geometry provides better matched confinement for bubble escape and wall rewetting, facilitating sustained liquid-film renewal and two-phase disturbance and thus maintaining a higher effective heat transfer contribution.

Figure 8c,d present the HTC–$q^{\prime \prime }$ and $q^{\prime \prime }$–ΔT relationships for CF (500 PPI) under different AR, where the trend is more evident: AR = 1.0 significantly outperforms AR = 0.7 and AR = 1.3 in the mid-to-high heat flux range and simultaneously shows the advantages of high HTC and low superheat requirement. For example, AR = 1.0 achieves *h* of about 102.6 kW/(m^2^ · K) at $q^{\prime \prime }$ of about 227.2 W/cm^2^ with ΔT of about 23.3 K. In comparison, AR = 0.7 yields *h* of about 72.5 kW/(m^2^ · K) at $q^{\prime \prime }$ of about 212.5 W/cm^2^ and requires a higher ΔT of about 29.3 K. AR = 1.3 performs the worst: at $q^{\prime \prime }$ of about 223.3 W/cm^2^, *h* is only about 42.0 kW/(m^2^ · K), and ΔT rises to about 44.3 K, indicating a larger equivalent thermal resistance. In addition, AR =1 achieves a limiting heat flux of 348.6 W/cm^2^, corresponding to an HTC of 55.4 kW/(m^2^ · K), and a limiting HTC of 130.3 kW/(m^2^ · K). Taken together, these curves suggest that when AR is too small (wider and shallower), confinement is insufficient: bubbles can escape prematurely, but sustained liquid replenishment and interfacial renewal are not sufficiently strengthened. When AR is too large (narrower and deeper), vapor retention and restricted venting are more likely, and higher superheat is required to reach the same heat flux. Therefore, AR = 1.0 provides a better balance among controllable bubble growth, smooth vapor venting, and rapid liquid replenishment.

**Figure 9:**
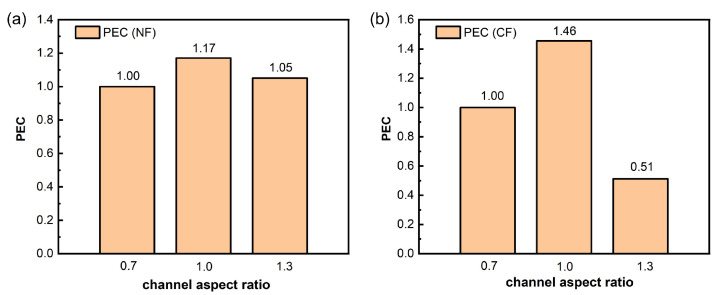
(**a**) PEC for NF under different AR (AR =0.7 represents the standard condition). (**b**) PEC for CF under different AR (AR =0.7 represents the standard condition).

To further demonstrate the comprehensive advantage of AR = 1.0, Figure 9 compares the PEC of 500 PPI grooved metal foams under different channel aspect ratios (AR = 0.7, 1.0, and 1.3). Taking AR = 0.7 as the reference (PEC = 1.0), the PEC for NF (PEC–NF) increases to 1.17 at AR = 1.0 and is 1.05 at AR = 1.3, indicating that AR = 1.0 provides a better balance between heat transfer gain and flow resistance penalty. For CF (PEC–CF), the trend is more pronounced: PEC reaches 1.46 at AR = 1.0, but drops to 0.51 when AR increases to 1.3, indicating a significant degradation in overall performance. According to the PEC definition, these results imply that when AR is too large, although local heat transfer may still be enhanced to some extent, the increased flow resistance and higher risk of vapor retention and local blockage cause the pressure drop penalty to dominate the comprehensive evaluation, especially for CF, where PEC decreases sharply. In contrast, AR = 1.0 achieves a strong balance between boiling heat transfer and flow resistance, yielding the highest PEC for both materials.

**Figure 10:**
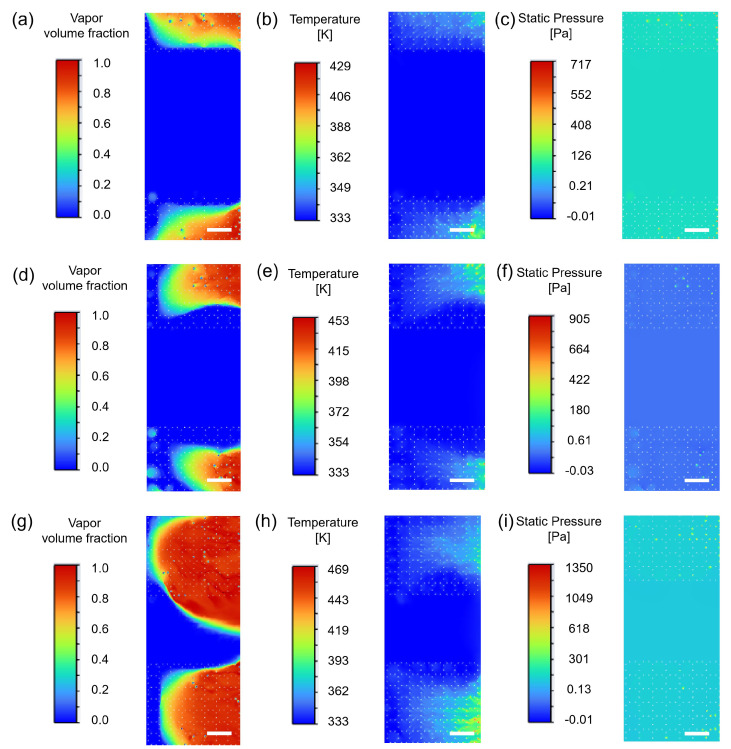
Vapor volume fraction, temperature, and pressure contours under different ARs. (**a**) Vapor volume fraction at AR = 0.7; (**b**) temperature at AR = 0.7; (**c**) pressure at AR = 0.7; (**d**) vapor volume fraction at AR = 1; (**e**) temperature at AR = 1; (**f**) pressure at AR = 1; (**g**) vapor volume fraction at AR = 1.3; (**h**) temperature at AR = 1.3; and (**i**) pressure at AR = 1.3. Scale bar is 0.3 mm.

To further elucidate the coupled influence of AR on vapor–liquid distribution, temperature field, and flow resistance in 500 PPI grooved metal foams, transient two-phase flow boiling simulations were carried out for AR = 0.7, 1.0, and 1.3. The results are shown in Figure 10a–i. From the vapor volume fraction contours, AR = 0.7 (Figure 10a) exhibits a large region of high vapor volume fraction clustered near the channel downstream end, suggesting vapor accumulation in the channel rather than being effectively guided out; such local vapor occupation weakens continuous liquid renewal to the channel and foam ligaments. AR = 1.0 (Figure 10d) shows a more controlled vapor morphology: the high-vapor region mainly forms a connected venting path along the flow direction while its lateral expansion is limited, and a continuous liquid pathway is retained in the channel core. This indicates that AR = 1.0 can ensure vapor-venting connectivity while maintaining effective liquid replenishment and rewetting. In contrast, AR = 1.3 (Figure 10g) forms larger contiguous high vapor coverage regions within the channel, with more pronounced vapor intrusion into the cross section, reflecting a stronger blockage tendency unfavorable for stable two-phase organization and continuous replenishment.

These differences are directly supported by the temperature and pressure contours. For AR = 0.7 (Figure 10b), a more prominent local temperature rise is observed in regions corresponding to downstream vapor clusters, indicating that vapor spreading and limited rewetting can increase hotspot risk. The pressure distribution (Figure 10c) does not show globally high pressure, but local pressure disturbances are more likely in vapor-rich regions. For AR = 1.0 (Figure 10e), the temperature field is more uniform with a smaller hotspot area, suggesting more sufficient alternating vapor–liquid contact and more stable thermal boundary-layer renewal; meanwhile, the pressure distribution (Figure 10f) exhibits a smoother pressure gradient, reflecting better matching between geometric confinement and vapor–liquid transport. AR = 1.3 (Figure 10h,i) shows more extended high-temperature regions and a more unfavorable pressure response: the narrow and deep channel increases flow resistance, and the increased vapor occupation further elevates two-phase pressure drop and restricts vapor venting, ultimately weakening rewetting and degrading boiling stability. Overall, AR = 1.0 achieves a better balance among venting smoothness, replenishment accessibility, and flow-resistance cost, providing mechanistic support for the experimentally observed higher HTC and lower superheat penalty. It should be emphasized that the superiority of AR =1.0 reported here is identified within the tested parameter space and operating conditions of this study; therefore, AR =1.0 should be regarded as a case-specific design reference rather than a universal optimum.

**Figure 11:**
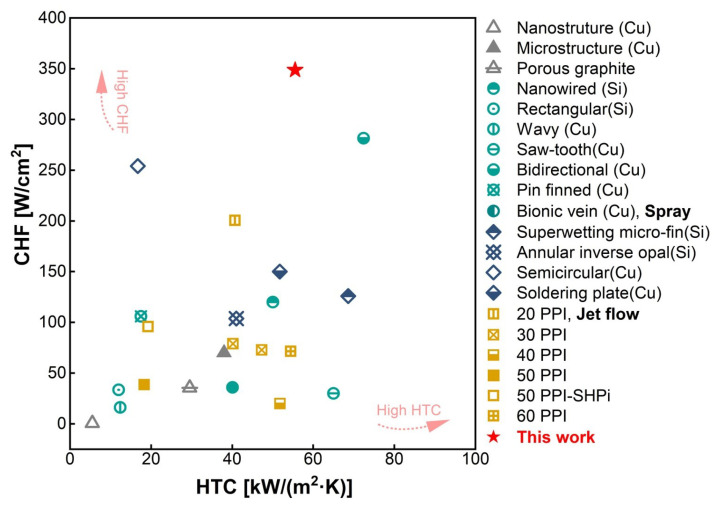
Comparative analysis of HTC and CHF in previous studies [32,33,34,35,36,37,38,39,40,41,42,43,44,45,46,47,48,49,50,51].

The CHF–HTC comparison (Figure 11) further highlights the overall advantage of the proposed CF evaporators in achieving a synergistic improvement in both heat transfer coefficient and critical heat flux. In particular, the data point of the 500 PPI CF with AR = 1 falls into the high-performance region of the map. By plotting the measured CHF against the corresponding HTC for various boiling-enhancement structures reported in the literature, it is evident that a trade-off between HTC and CHF is commonly observed in conventional boiling enhancement: structures that markedly increase HTC by intensifying nucleation activity and interfacial disturbance are often more prone to vapor blanketing or local dryout, thereby limiting further CHF enhancement; conversely, configurations that effectively delay dryout and achieve higher CHF may provide insufficient heat transfer intensification, resulting in relatively lower HTC. Specifically, the bidirectional counter-flow microchannels [32] or wettability-treated surfaces [33] can deliver relatively high HTC values (>$60\;\mathrm{kW}/(m^{2}\cdot K)$); yet, their CHF is typically below $300\;W/{\mathrm{cm}}^{2}$, and the HTC may even decrease before reaching CHF. In contrast, the surface of the ring-shaped chalcedony can achieve higher CHF, but usually at the expense of a lower HTC [34]. Overall, most conventional configurations cluster in the region of HTC $<50\;\mathrm{kW}/(m^{2}\cdot K)$ and CHF $<150\;W/{\mathrm{cm}}^{2}$. By comparison, the data points of the present CF evaporators shift toward the “high HTC–high CHF” quadrant, indicating that they can maintain strong heat transfer performance, thereby delivering superior overall boiling performance relative to most reported studies.

## 5. Conclusions

Experiments and simulations were conducted to compare the flow boiling heat dissipation performance of NF and CF. For ungrooved metal foams, the effects of pore density (100, 500, and 1000 PPI) were analyzed; for grooved metal foams, the effects of channel aspect ratio (0.7, 1.0, and 1.3) were evaluated in terms of HTC, ΔT, and PEC. Simulations were used to validate experimental trends and to provide preliminary mechanistic insights. The main conclusions are as follows:

(1) Non-monotonic pore-density effect, with 500 PPI as the optimal pore density. For both NFs and CFs, increasing pore density from 100 to 500 PPI significantly improves HTC and ΔT response, indicating that a larger specific surface area and more reasonable pore connectivity enhance two-phase disturbance and interfacial renewal. However, further increasing pore density to 1000 PPI limits overall performance due to increased flow resistance and a higher risk of local blockage. Numerical simulations further verify this mechanism: 100 PPI tends to form larger vapor clusters and induces local hotspots and pressure disturbances, 1000 PPI produces more distributed vapor structures, but pore-scale confinement and permeability resistance lead to higher pressure drop and restricted liquid replenishment. In contrast, 500 PPI more readily forms connected yet non-blocking vapor-venting paths, with a more uniform temperature field and a more controllable pressure drop. PEC comparisons also support this conclusion: relative to the 100 PPI baseline (PEC = 1), the PECs of 500 PPI NFs and CFs increase to 1.24 and 1.31, whereas those of 1000 PPI metal foams decrease to 0.93 and 0.73.

(2) The channel aspect ratio (AR) governs the trade-off between heat transfer enhancement and flow resistance, with an optimal value of AR =1.0 identified. For grooved 500 PPI samples, AR =1.0 yields a higher heat transfer coefficient (HTC) and a more favorable temperature difference (ΔT) for both material systems, indicating an improved balance among smooth vapor venting, efficient bubble escape, and accessible liquid replenishment. At AR =0.7, insufficient geometric confinement causes vapor to remain spatially dispersed, thereby weakening directed vapor venting and rewetting efficiency. In contrast, increasing the AR to 1.3 elevates vapor-venting resistance due to overly narrow and deep channels, increasing the likelihood of vapor retention, amplifying temperature non-uniformity, and deteriorating the pressure drop response. The simulation results are consistent with experimental observations: AR =1.0 promotes the formation of continuous vapor pathways without large-scale vapor coverage, suppresses hotspot expansion, and maintains smoother pressure gradients, whereas AR =1.3 more readily exhibits vapor cross-sectional occupation and an increased pressure drop. The performance evaluation criterion (PEC) further confirms the comprehensive advantage of AR =1.0, with values of 1.17 for NF and 1.46 for CF. By contrast, the PEC of CF at AR =1.3 drops sharply to 0.51, indicating that excessively narrow or deep channels significantly exacerbate flow resistance and operational instability.

(3) Optimal configuration and key performance metrics. Based on the overall experimental results, the grooved metal-foam evaporator with 500 PPI and AR =1 achieved a limiting heat flux of 348.6 W/cm^2^, corresponding to an HTC of 55.4 kW/(m^2^ · K), and a limiting HTC of 130.3 kW/(m^2^ · K). This demonstrates that synergistic matching of pore density and channel aspect ratio can markedly enhance both heat transfer capability and stability in the high heat flux regime, providing direct guidance for the design of evaporators for high-heat-flux electronics cooling.

These results provide a theoretical basis for designing next-generation high-efficiency cooling systems for high-power-density electronic devices (especially edge data centers). It should be emphasized that the optimal PPI and AR reported here are valid only within the tested parameter space (PPI = 100–1000, AR =0.7–1.3) and the present operating conditions (deionized water at 60 °C, atmospheric pressure, bottom heating). The optimum may shift with changes in working fluid, mass flux, inlet subcooling, system pressure, or heating boundary conditions; therefore, these values should be treated as case-specific design references rather than universal optima.

Despite the high HTC and CHF achieved, the present results reveal remaining bottlenecks, including the elevated pressure drop penalty and stability risk at high heat fluxes, insufficient vapor–liquid separation leading to vapor accumulation or flow maldistribution, the case-dependent nature of the identified optimal PPI or AR, and the limitations of the 2D VOF equivalent foam model in capturing true 3D connectivity and tortuosity. Accordingly, future work should couple foam or groove optimization with advanced wettability engineering (e.g., patterned or gradient wettability) and multiscale groove architectures (e.g., hierarchical separation channels and manifold-assisted distribution) to enhance rewetting and vapor venting while suppressing maldistribution under ultra-high heat fluxes. In addition, integrating spray or jet-impingement cooling and developing more realistic 3D-resolved models are recommended to further extend CHF and HTC and improve quantitative predictions of pressure drop and phase distribution.

## Figures and Tables

**Table 1:** Structural parameters.

$H_{\mathrm{ch}}/W_{\mathrm{ch}}$	$W_{\mathrm{cel}}$ (mm)	$L_{\mathrm{eva}}$ (mm)	$H_{\mathrm{water}}$ (mm)	$H_{\mathrm{CF}}$ (mm)	$H_{\mathrm{eva}}$ (mm)	$H_{u}$ (mm)
0.7/1.0/1.3	1.4	30	0.5	1.0	1.5	5

## Data Availability

The data presented in this study are available on request from the corresponding author.

## Supplementary Materials

The following supporting information can be downloaded at: https://www.mdpi.com/article/10.3390/mi17030286/s1.
