# Supplementary material for: Investigation of Flow Boiling Heat Transfer Performance of Grooved Metal Foam (Ni, Cu) Evaporators
Source: Micromachines (Basel). 2026 Feb 25;17(3):286. doi: 10.3390/mi17030286 (PMC13028134; doi:10.3390/mi17030286)
Supplement: Supplementary file 1 [file micromachines-17-00286-s001.zip › micromachines-4151900-supplementary.pdf]

Supplementary Information for:

Investigation on Flow Boiling Heat Transfer Performance of grooved Metal Foam (Ni, Cu) Evaporators

Junteng Cao[a], Huajie Li[a], Xianbo Nian[a],
Chaoyi Zhang [a], Yuankun Zhang[a] , Chunsheng Guo[a*]

[a] Centre for Omniscale Thermal Management and Comprehensive Energy Utilisation (OTM-EU), School of Airspace Science and Engineering, Shandong University, Weihai, 264209, China

E-mail: C. Guo: guo@sdu.edu.cn

Contents

Supplementary Note 1 | The detailed parameters and process flow for wire cutting of foam metal using electric discharge machining.
Supplementary Note 2 | Detailed welding process and description of thermal resistance at the welding point.
Supplementary Note 3 | Discussion on Pressure drop Δp.
Supplementary Note 4 | Simulation settings.

Supplementary References

Supplementary Note 1 | The detailed parameters and process flow for wire cutting of foam metal using electric discharge machining.

The foam metal minichannels were fabricated by wire electrical discharge machining (WEDM) using a Sodick wire-cut EDM system (labeled “Wire-cut EDM”, model ALN400Qs). The machine integrates a wire-feeding unit, guide rollers/guiding assemblies, and a CNC worktable, and the machining zone is located inside a dielectric bath. Material removal is achieved via pulsed electrical discharges across a small inter-electrode gap between the wire electrode and the workpiece. The dielectric fluid simultaneously provides electrical insulation, cooling, and debris evacuation, thereby stabilizing the discharge process and reducing the risk of secondary discharges. Deionized water was used as the dielectric, with conductivity maintained at 5-15 μS/cm. A brass wire with a diameter of 0.10 mm served as the wire electrode. To ensure discharge stability and wire-path rigidity, the wire speed and wire tension were set to 6-10 m/min and 6-10 N, respectively. The flushing strategy primarily relied on immersion in the dielectric bath with mild flushing; the nozzle/side flushing pressure was controlled at 0.08-0.20 MPa to balance debris removal while preventing collapse of the foam skeleton.

During machining, the foam metal specimen was uniformly supported by a fixture and clamped on the worktable. The groove profile was generated by scanning the contour with the fine wire electrode. Considering the thin skeleton of CF and its susceptibility to erosion and slag attachment, a two-step strategy (“rough cutting + low-energy trimming”) was adopted. The rough cut prioritized geometric fidelity and machining efficiency, with typical electrical parameters of an open-circuit voltage of 70-90 V, peak current of 2-4 A, pulse-on time T_{on} = 1.0-2.0 μS, and pulse-off time T_{off} = 8-15 μS; the servo gap (Servo/SV) was moderately increased to suppress short circuiting and arcing. Subsequently, one low energy trim pass was applied to improve the groove-wall quality and reduce the recast layer, during which the discharge energy was lowered to a peak current of 1-2 A, T_{on} = 0.5-1.5 μS, and T_{off} = 12-20 μS.

Supplementary Note 2 | Detailed welding process and description of thermal resistance at the welding point.

First, the solder paste is applied to the top surface of the heating block, which is a 3 cm × 2 cm square. Next, the cartridge heaters are powered using a DC power supply to heat the solder paste to 237 °C, melting the solder layer. Finally, the CF sample is pressed onto the molten solder layer with a weighted load until the temperature returns to room temperature, completing the bonding process.

The thermal contact resistance at the interface between the copper foam and the OFC heating block may affect the back-calculated wall temperature and HTC. In this work, the foam sample was metallurgically bonded to the OFC block using a high-temperature lead-free solder paste (Sn99Ag0.3Cu0.7), forming a thin and continuous solder layer. Therefore, heat transfer across the interface is dominated by bulk conduction through the solder rather than by discrete micro-contact conduction, and the interfacial thermal resistance is expected to be negligible. Compared with a non-bonded (simply placed) dry contact, metallurgical soldering forms a thin, continuous conductive layer and increases the interfacial thermal conductance by approximately 100 times. A conservative estimate yields an effective interfacial thermal resistance of about $10^{-6}\ \mathrm{m^2 \cdot K/W}$ or lower, corresponding to only a minor additional temperature drop under the present heat flux range. Moreover, all samples were prepared using the same solder material and bonding procedure; thus, any residual interfacial resistance would mainly introduce a small systematic offset and does not alter the comparative trends and conclusions regarding the effects of PPI and groove geometry.

Supplementary Note 3 | Discussion on Pressure drop Δp.

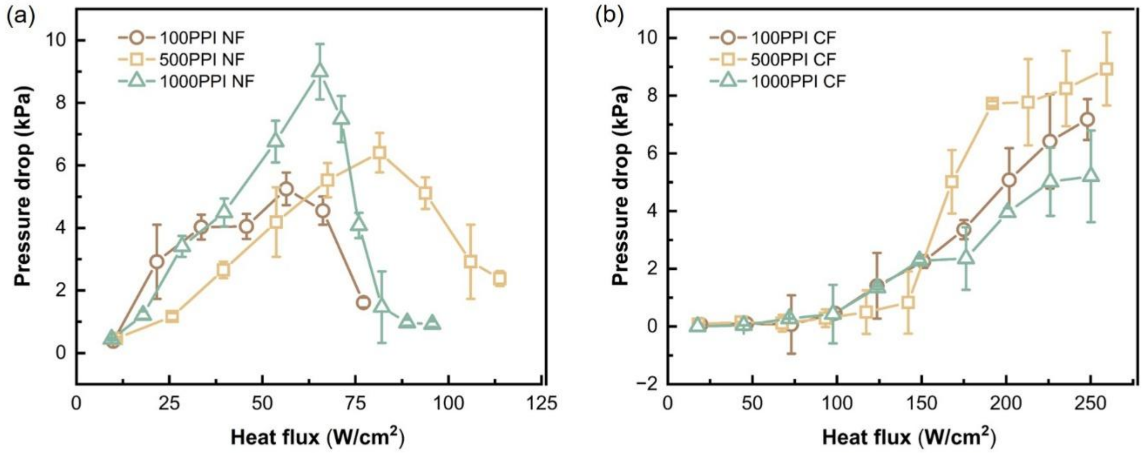

Figure S1 (a) Pressure drop-q" for NF at different PPI (b) Pressure drop-q" for CF at different PPI.

From the perspective of pore density (PPI), the two-phase pressure-drop comparison (Fig. S1 a-b) shows that, for the non-grooved NF configurations, the pressure drop exhibits a typical "increase–decrease" behavior with increasing heat flux. As the heat flux rises from low values and the boiling-enhanced regime is entered, vapor generation rapidly increases both the frictional and accelerational components, leading to a progressive rise in Δp and a peak at intermediate heat fluxes. Among the NF samples, the 1000 PPI case yields the highest peak ($\approx$9 kPa), while the peak values for 500 PPI and 100 PPI are approximately 6 kPa and 5 kPa, respectively. At higher heat fluxes, Δp decreases markedly, indicating that, as the flow pattern evolves toward a more continuous vapor-core/annular-like structure, the contribution of two-phase mixture friction and intermittent slug-like structures to the overall pressure drop is mitigated. In contrast, the grooved CF configurations display an overall monotonic increase in Δp with heat flux: Δp remains close to zero in the low-heat-flux regime dominated by single-phase flow or weak boiling, then increases rapidly once a pronounced two-phase regime is established (approximately 140–170 W/cm²), and remains relatively high at elevated heat fluxes (e.g., the 500 PPI CF reaches ~7–9 kPa in the 200–250 W/cm² range). The error bars represent the dispersion of the pressure-drop signal under quasi-steady conditions; while the dispersion increases with boiling intensity, no divergent jump behavior is observed.

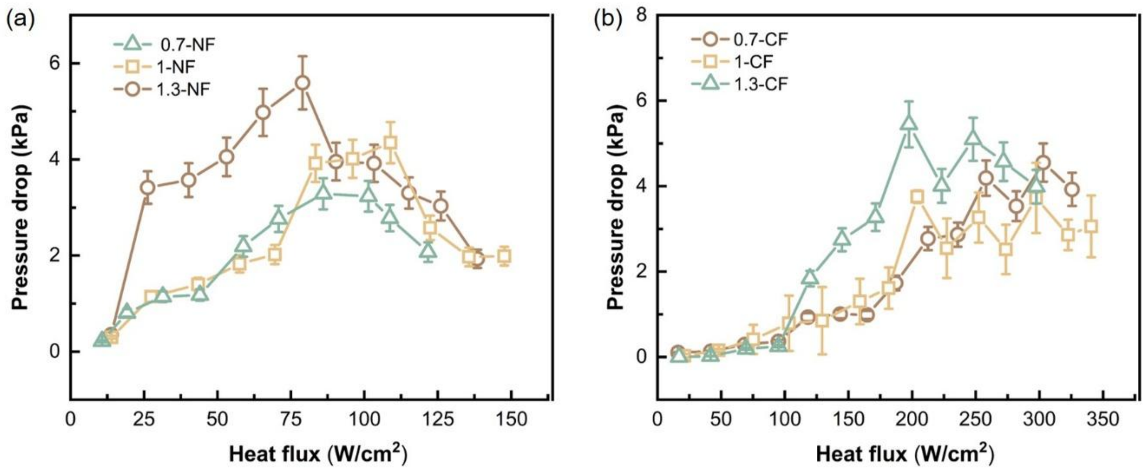

Figure S2 (a) Pressure drop-q" for NF at different AR (b) Pressure drop-q" for CF at different AR.

Further analysis from the standpoint of groove aspect ratio (AR) (Fig. S2 a-b) indicates that, for the NF configurations, increasing AR generally results in a higher pressure-drop level and a more pronounced peak. For example, at intermediate heat fluxes, the Δp for AR = 1.3 increases to approximately 5–6 kPa, which is significantly higher than those for AR = 0.7 and AR = 1. This suggests that stronger geometric confinement enhances two-phase frictional resistance and the additional pressure-drop contributions associated with intermittent vapor slugs and bubble coalescence. At higher heat fluxes, Δp similarly decreases, reflecting a change in the dominant pressure-drop mechanism as the flow pattern transitions. For the CF configurations, the effect of AR is mainly manifested as differences in Δp and local peaks in the high-heat-flux regime: with increasing heat flux, Δp generally rises and remains in the range of about 3–6 kPa over approximately 200–300 W/cm^2, with discernible differences among the AR cases. This indicates that the groove geometry influences the pressure drop level and fluctuation amplitude by modifying vapor venting and liquid replenishment pathways.

Supplementary Notes 4 | Simulation settings.

The simulation model is a two-dimensional cross-section of foam metal. Different pore densities are simulated by setting different skeleton sizes (indicated by

the blank circles in the figure) and the distances between the skeletons, and the depth-to-width ratio of the channels is varied to achieve different ratios.

The gas–liquid interface was tracked using the volume-of-fluid (VOF) method. Phase change was modeled via a Lee-type mass source term, and surface tension was treated using the continuous surface force (CSF) formulation. For boundary conditions, an inlet velocity condition with v_{in} (66 kg·m^{-2}·s^{-1}) at T_{in} (333.15 K), and the outflow condition were applied at the inlet and outlet, respectively. A constant heat flux q'' (200 W/cm^2) was imposed on the bottom heated wall, whereas the remaining outer walls were treated as adiabatic. Due to the two-dimensional model, the channel depth remains unchanged. Therefore, the channel widths in these three cases (AR = 1.3, 1.0, 0.7) are set to 1.0, 1.3, and 1.9 millimeters respectively. The governing equations were solved using the SIMPLE pressure–velocity coupling scheme, with pressure discretized using PRESTO! method. The second-order upwind was applied for the iteration of the energy governing equation. The time step was set to Δt=10^{-4}s to ensure numerical stability for interface evolution and phase-change calculations.
